# MIST: A microbial identification and source tracking system for next‐generation sequencing data

**DOI:** 10.1002/imt2.146

**Published:** 2023-11-02

**Authors:** Minghui Song, Chang Han, Linmeng Liu, Qiongqiong Li, Yiling Fan, Hao Gao, Dan Zhang, Yi Ren, Feng Qin, Meicheng Yang

**Affiliations:** ^1^ Shanghai Institute for Food and Drug Control NMPA Key Laboratory for Testing Technology of Pharmaceutical Microbiology Shanghai; ^2^ Shanghai Majorbio Bio‐Pharm Technology Co., Ltd. Shanghai China; ^3^ Shanghai food and drug packaging material control center Shanghai China

## Abstract

The Professional Committee of Microbiology of the National Pharmacopoeia Commission organized the drafting of the Technical Guidelines for Microbial Whole Genome Sequencing (WGS), aiming to standardize the method process and technical indicators of microbial WGS and ensure the accuracy of sequencing and identification. On the basis of the Guidelines, we developed an integrated microbial identification and source tracking (MIST) system, which could meet the needs of microbial identification and contamination investigation in food and drug quality control. MIST integrates three analysis pipelines: 16S/18S/internal transcribed spacer amplicon‐based microbial identification, WGS‐based microbial identification, and single‐nucleotide polymorphism‐based microbial source tracking. MIST can analyze sequence data in a variety of formats, such as Fasta, base call file, and FASTQ. It can be connected to a high‐throughput sequencing instrument to acquire sequencing data directly. We also developed a publicly accessible web server for MIST (http://syj.i-sanger.cn).

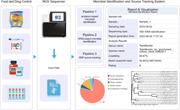

Microbial identification is of great value for clinical, epidemiological, food, and pharmaceutical research [1]. Traditionally, microbes have been identified based on their morphological, physical, and biochemical properties [2]. However, many prokaryotic microbes are difficult to culture using traditional methods [3] and thus cannot be detected by traditional methods. These unculturable microbes harbor a potential source of novel metabolites and are essential components of natural metabolic networks [4]. Moreover, traditional methods also fail to detect novel culturable microbes and have problems in detecting unusual microbes that have not been comprehensively evaluated [5]. High‐throughput sequencing technology (HTS) has enabled sequence‐based genomics to become one of the routine and promising methods for microbial identification [6]. HTS‐based methods can be subdivided into two categories: amplicon sequencing [7], which amplifies conserved sequences in microbes (e.g., 16S ribosomal RNA [rRNA] for bacteria and 18S recombinant DNA [rDNA]/internal transcribed spacer [ITS] region for fungi), and whole genome sequencing (WGS) [8], which sequences the whole genomes of a microbe after isolation. The 16S rDNA‐based amplicon sequencing is an efficient method to investigate all bacteria in a sample because this region has been recognized as the conventional method for prokaryotic identification [9]. The community has accumulated a large amount of well‐characterized 16S rDNA sequences in large databases, such as Ribosomal Database Project [10] and SILVA [11]. Amajor limitation of amplicon sequencing is its lack of discrimination among closely related species [12]. WGS‐based bacterial identification provides higher discriminatory power and allows bacterial identification at species or even at strain level. It also provides a powerful way for investigating functional genes, such as antibiotic resistance genes (ARGs) [13, 14] and virulence factors genes (VFGs) [15]. Furthermore, the multilocus sequence type (MLST) [16] and single‐nucleotide polymorphism (SNP) [17, 18, 19] enable source tracking of genetically closely related bacteria that were isolated from different sources. Such analysis enables WGS‐based applications in multiple fields, such as forensic investigations, strain identification, and outbreak tracking [20].

Currently, there are some web services and tools for microbial identification, for example, BacWGSTdb [21], ImageGP [22], Bacterial Analysis Pipeline (CGE) (https://cge.cbs.dtu.dk/services/cge/) [23], Qiime2 [24], EasyAmplicon [25], GCType (GCM Type Strain Sequencing project), and rANOMALY [26]. Each website has its own unique strengths and limitations. For example, BacWGSTdb offers MLST‐based and whole‐genome‐based bacterial genotyping but only accepts assembly genome files as inputs. CGE provides various tools for genome‐based phenotyping, phylogeny, and annotation of ARGs and VFGs. However, users should upload their data into FASTQ each of these tools separately due to the lack of an integrated backend. Furthermore, all web‐based tools require a fast and consistent internet connection to upload raw sequence files, which can have sizes of hundreds to thousands of MBs [8]. With the development of NGS technology, the downstream bioinformatics analysis is challenging, and more software and systems need to be developed [27, 28].

Here, we present a system for the classification and identification of microbes. It implements sophisticated pipelines for both amplicon sequencing data, which enable efficient profiling of unculturable microbes, and WGS data, which enable accurate genotyping of cultured microbes. The system also implements pipelines for the MLST, SNP‐based source tracking, and ARGs or VFGs annotation from WGS data.

## RESULTS

### Overview of the system

**Figure 1:**
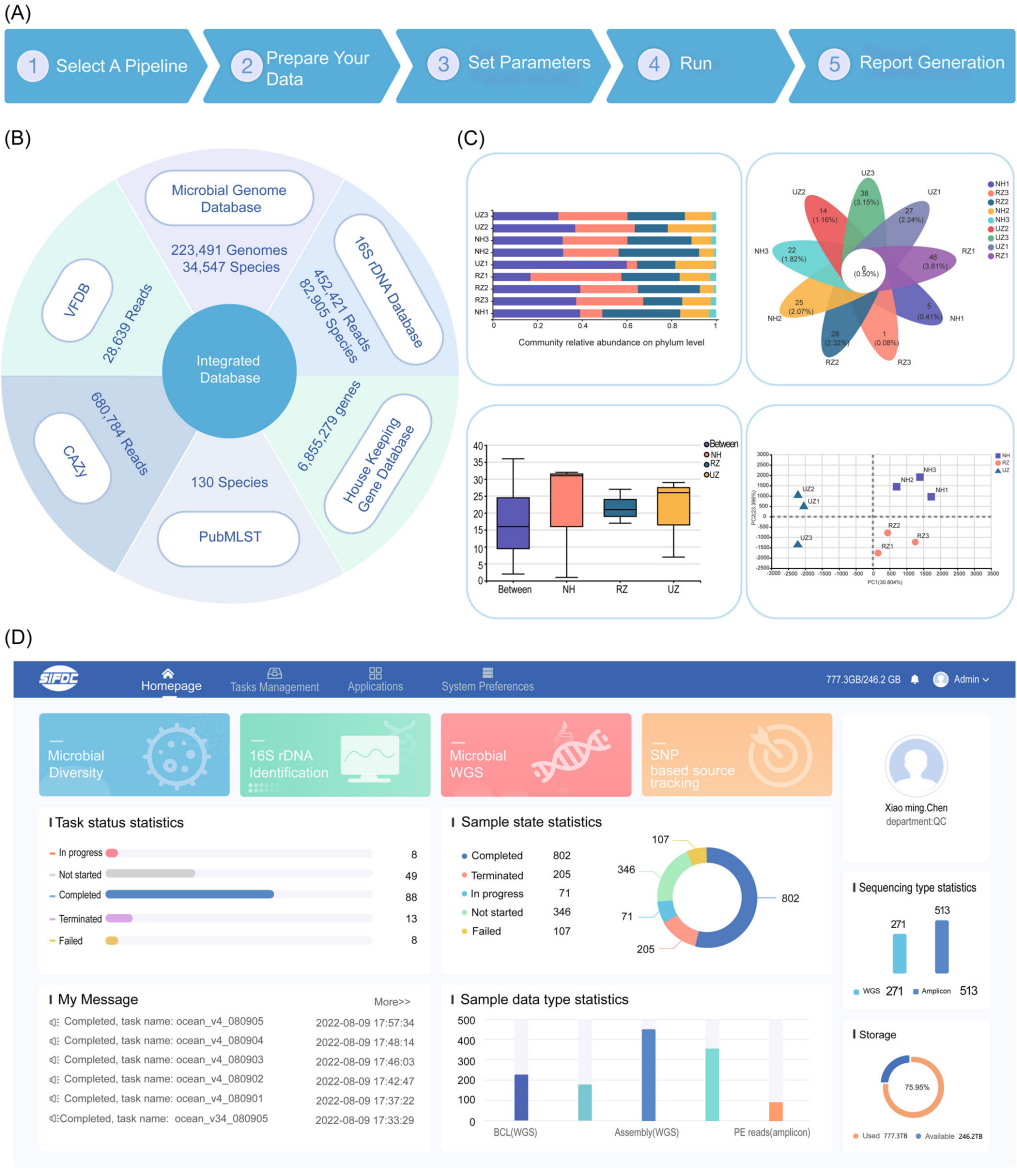
Overview of the MIST system. (A) Five data analysis steps implemented in the MIST. (B) Integrated databases included in MIST. (C) Examples of data visualization. (D) Home Page of the MIST web server. CAZy, carbohydrate‐active enzymes; MIST, microbial identification and source tracking; PE, paired end; rDNA, recombinant DNA; SNP, single‐nucleotide polymorphism; VFDB, virulence factor database; WGS, whole genome sequencing.

The system consists of three pipelines: (1) amplicon‐based microbial identification, such as 16S rDNA/18S rDNA/ITS genes, (2) WGS‐based microbial identification, and (3) SNP‐based source tracking. To initiate the analysis, users only need to choose sequencing files in base call file or FASTQ format generated by Illumina sequencer, or Fasta‐formatted sequence files (such as assembled genomes or 16S sequences) into the server. Then, users can create a task by selecting a pipeline and setting corresponding parameters. Finally, sequencing data and parameters are submitted to the server and trigger the analytic pipelines (Figure 1A). The system provides mainstream reference databases for microbial identification and functional annotation (Figure 1B). We also have a data management system that is responsible for monitoring the processing tasks and managing the database, such as inputs and outputs files (Figure 1D). Users can view the task results on the online interactive analysis report interface and download the results for further use (Figure 1C).

### Pipeline 1: 16S rDNA/18S rDNA/ITS amplicon‐based microbial identification

**Figure 2:**
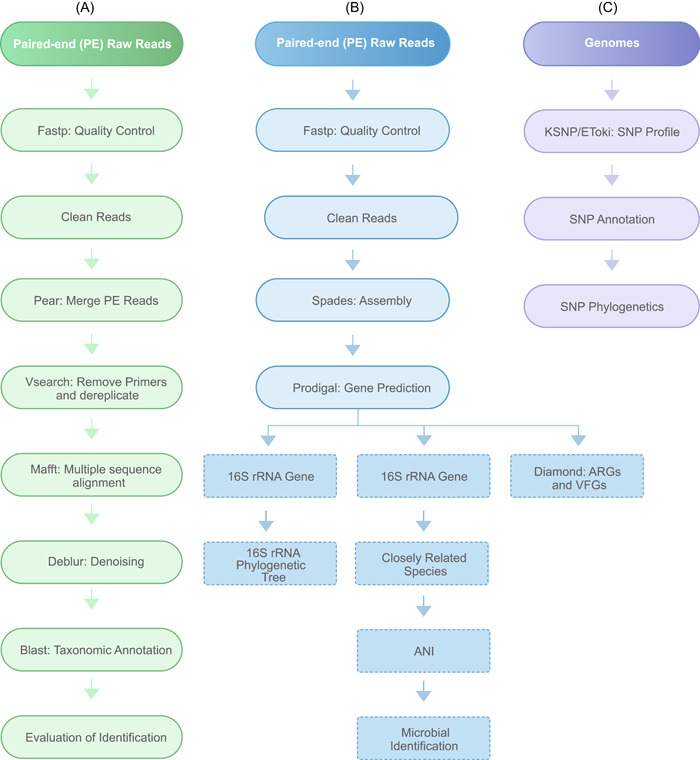
Workflow of three pipelines. (A) Amplicon‐based microbial identification, such as 16S rDNA/18S rDNA/ITS genes. (B) SNP‐based source tracking. (C) WGS‐based microbial identification. ANI, Average Nucleotide Identity; ARGs, antibiotic resistance genes; rDNA, recombinant DNA; rRNA, ribosomal RNA; SNP, single‐nucleotide polymorphism; VFGs, virulence factors genes; WGS, whole genome sequencing.

This pipeline can be used to identify microbes, cultured or uncultured, using 16S/18S rDNA and ITS regions. The pipeline contains “Quality Control,” “Primer Removal,” “Denoising,” “Annotation,” and “Evaluation” functional components. In short, Fastp v0.23.4 [29] was used to perform quality control and clean the paired‐end (PE) FASTQ reads by trimming and filtering reads based on their quality and length. The reads were truncated at any site receiving an average quality score of <20 over a 50 bp sliding window, and the truncated reads shorter than 50 bp were discarded; reads containing ambiguous characters were also discarded. The resulting reads were subjected to the server for merging the pair‐end reads, followed by primer removal by a homemade Python script, duplicate removal by vsearch v2.22.1 [30], and denoising by deblur v1.1.1 [31]. The procedure above generates a set of amplicon sequence variants (ASVs), which were each treated as a taxonomic unit. Each ASV was then aligned to a reference genome database using BLASTn v2.11.0 [32]. The taxonomic classification of ASV was estimated by best‐hits matches in the reference database. Phylogenetic tree was constructed by the maximum likelihood (ML) method. The workflow is illustrated in Figure 2A.

We selected dozens of bacterial species from two different habitats, the human gut and marine, and generated corresponding simulated sequencing data based on the V3–V4, V4, and V4–V5 regions of 16S ribosomal gene. On the basis of the simulated data, the performance of the amplicon identification program was tested. All the bacteria were identified correctly on the genus level (Table S1).

### Pipeline 2: WGS‐based microbial identification

The WGS has been increasingly used in basic research and clinical diagnostics. In our system, we used housekeeping genes and Average Nucleotide Identity (ANI) to identify microbial species and infer their phylogenetic relationships with others. The pipeline contains six modules: Quality control, Assembly, Gene prediction, ANI calculation, Annotation, and MLST.

Fastp was used for quality control and cleaning the PE FASTQ reads. In the assembly process, SPAdes v3.11 [33] was used to assemble the genome, but for some contaminated samples, the metaSPAdes v3.10 [34] was used for contaminated sample assembly. BUSCO v5.1 [35] was used to evaluate the completeness and contamination of the genomes.

We used Prodigal to predict the open reading frames and then translated them into protein products. HMMER v3.1b [36] was used to find the 31 single‐copy housekeeping genes (for genes list, see genome database curation) in the genome. The databases CARD v3.1.3 and carbohydrate‐active enzymes (CAZy) (202001 updated) [37] were used separately to identify the possible ARGs and CAZy, with the parameter of *e*‐value > 1e − 5. The database virulence factor database (VFDB) 2022 is used to identify potential virulence factors for the identified pathogen strain.

The strategy for bacterial identification was as follows:

1. Extracting the sequences of the single‐copy housekeeping genes from predicted genes after HMM search against 31 single‐copy housekeeping genes profiles.
2. Blasting each of the housekeeping genes against the 31 single‐copy housekeeping genes database and keeping the top 200 blast results for each gene under *e*‐value > 1e − 5 with the same score and identity. For each species in the database, we then counted the number of housekeeping genes that included the species in the blast results and ranked species based on the number. By default, the pipeline filtered out the species with the counted number of housekeeping genes less than 15, but this value can be modified by users. So our strategy can identify not only the cultured individual microbes but also the contaminating samples.
3. The ANI value was calculated between the genome of the sample and each genome of the species selected from the above method, and only the maximum ANI value of a species was reported. For some species that contained too many strains, we chose up to 1000 strains for ANI calculation.
4. Barrnap v0.9 (https://github.com/tseemann/barrnap) was used to predict 16S rDNA. The phylogenetic tree of 16S rDNA and housekeeping genes was built using IQ‐TREE v1.6.12 [38].

Further, if the species identified were included in the PubMLST database (http://pubmlst.org) [39], the molecular typing of the sample was analyzed automatically. The workflow is illustrated in Figure 2C.

**Figure 3:**
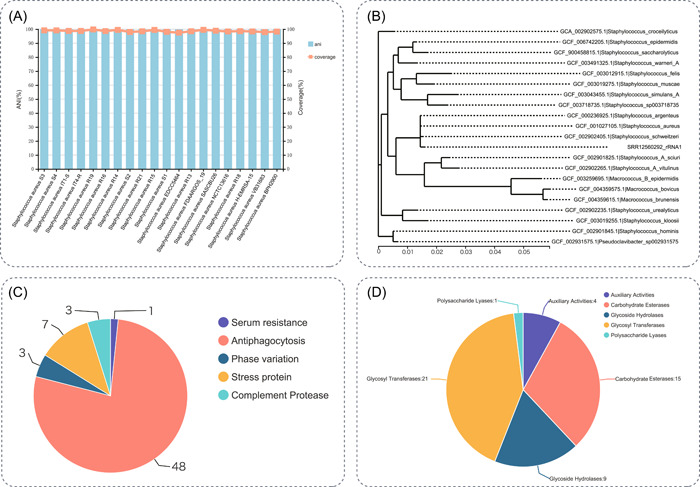
An example of microbial identification by WGS. (A) The ANI and coverage of strains. (B) A phylogenetic tree of 16S rDNA genes of the target strain and the related strains. (C) The statistics of virulent factor genes. (D) The statistics of CAZyme genes. NGS raw data with SRA accession number SRR12560292 was used. ANI, Average Nucleotide Identity; CAZyme, carbohydrate‐active enzyme; NGS, next‐generation sequencing; rDNA, recombinant DNA; SRA, Short Read Archive; WGS, whole genome sequencing.

This workflow was applied to analyze a sample, downloaded from the National Center for Biotechnology Information Short Read Archive database under accession number: SRR12560292. The sample data contained 1,418,820 reads, which produced 46 scaffolds, and the length of the assembly was 2.76 Mbp. The 31 single‐copy housekeeping genes were enriched in *Staphylococcus aureus*, and the *S. aureus* S3 was the most related strain in the database. The MLST type was ST22, and a total of 142 genes were identified as having a role in the resistance to various antibiotics in CARD and 462 virulence factors in this sample (Figure 3).

The genomes of 560 ATCC standard strains were downloaded to test the accuracy of our identification procedure. There were only five genomes whose identification results were inconsistent with their own names. Through careful analysis, it was found that three of them were caused by the naming error of the reference species in the database (GTDB database has corrected their names based on WGS). The other two had disputes about the nomenclature of the representative strains. However, all of our identifications came from the highest‐scoring genomes in the database (Tables S2–S4).

### Pipeline 3: SNP source tracking

In practice, in addition to microbial species identification, we also need to analyze the evolutionary relationship between different isolates of a certain species. For example, in a pharmaceutical factory environment, we can determine the source of strain contamination by analyzing the evolutionary distance between isolates.

Two modes for microbial traceability by SNP phylogeny are integrated into the system, which are implemented through the software EToki v1.2 [40] and kSNP v3.0 [18], respectively. In the EToki mode, SNPs are called by comparing genomes to a reference genome, and the derived consensus sequence file is used to create an ML phylogeny. The kSNP is a program for SNP identification and phylogenetic analysis without genome alignment or the requirement for reference genomes, which is more useful when the concerned microorganisms are unculturable or have a large intraspecies evolutionary distance. In addition, a phylogenetic tree view is provided in both modes. The workflow is illustrated in Figure 2B.

### Amplicon database and genome reference database

SILVA v138 and UNITE v8.0 [41] are integrated as the source of the amplicon reference database used in the microbial identification by 16S rDNA/18S rDNA/ITS pipeline and the microbial community diversity analysis pipeline. Details of the reference database are described in Table 1.

**Table 1:** Details of the reference database for amplicon sequencing‐based pipelines.

Species	Conserved region	Source	Number of sequences	Number of species
Bacteria and Archaea	16S rDNA	SILVA 138	452,421	82,905
Eukaryote	18S rDNA	SILVA 138	58,562	35,575
Fungi	ITS region	UNITE 8.0	887,397	33,032

Abbreviation: rDNA, recombinant DNA.

In addition, we built a housekeeping gene database covering 223,491 bacterial RefSeq [42] genomes for fast and accurate profiling of microbial identification in the WGS workflow. Genes with the same name or product of 31 single‐copy housekeeping genes (*dnaG*, *frr*, *infC*, *nusA*, *pgk*, *pyrG*, *rplA*, *rplB*, *rplC*, *rplD*, *rplE*, *rplF*, *rplK*, *rplL*, *rplM*, *rplN*, *rplP*, *rplS*, *rplT*, *rpmA*, *rpoB*, *rpsB*, *rpsC*, *rpsE*, *rpsI*, *rpsJ*, *rpsK*, *rpsM*, *rpsS*, *smpB*, and *tsf*) were extracted from each genome to construct the full database, which contains 6,855,279 amino acid sequences in total. The 31 single‐copy housekeeping genes database was used to identify probable species in the WGS pipeline.

## CONCLUSION

WGS, amplicon sequencing, and metagenomic sequencing are increasingly used in research to produce complicated environmental sequence data sets, which paved the way for a cultivation‐independent genetic content assessment and exploitation of the entire communities of organisms [4, 42, 43, 44]. Therefore, it is urgent to develop WGS and amplicon‐based microbial species identification pipelines in the field of food safety and drug control. Here, we provide a system to analyze the WGS, amplicon sequences for microbial identification, MLST typing, and SNP source tracking. In our system, one important potential use of the WGS microbial identification pipeline is to identify contaminated sequences or metagenome samples. Simultaneously, it has great value in speeding up pathogen detection in clinical laboratories, while the existing identification and taxonomy methods may be unreliable with contaminated samples.

## AUTHOR CONTRIBUTIONS

Meicheng Yang, Feng Qin, and Yi Ren conceived the system and idea. Linmeng Liu and Hao Gao implemented the MIST main code. Chang Han and Dan Zhang designed the graphical user interface. Minghui Song, Chang Han, and Linmeng Liu wrote the manuscript. Yi Ren, Chang Han, Qiongqiong Li, and Yiling Fan were responsible for editing and revising the manuscript. All authors contributed to the development of MIST.

## CONFLICT OF INTEREST STATEMENT

The authors declare no conflict of interest.

## Supporting information

Supporting information.

## Data Availability

Supplementary materials (tables, scripts, graphical abstracts, slides, videos, Chinese translated version, and update materials) may be found in the online DOI or iMeta Science http://www.imeta.science/.
